# Osteosarcoma and Encephaloid Tumour of the Fore-Arm

**Published:** 1844-05-18

**Authors:** 


### OSTEOSARCOMA AND ENCEPHALOID TUMOUR OF THE FORE-ARM.

A young countryman, who had upon two occasions received a severe kick from a horse on the right fore-arm, was admitted into the Hospital Saint Jean, at Brussels, having a tumour on the injured parts as large as a full-sized fœtal head, with three projections on it, where an obscure fluctuation, resembling that afforded by a gelatinous mass, could be felt, and where the skin was on the point of breaking. The largest of these was opened, and black clotted blood evacuated. By a slight degree of compression, several ounces of a black, shining, thick fluid were obtained from it, having masses of different size resembling melted fat, floating in it. The evacuation of this fluid was always followed by venous hemorrhage. Erysipelas and sphacelus next attacked the poor fellow, and the hospital surgeon deemed it requisite to perform amputation, which however failed in saving the patient's life. He died a fortnight afterwards. On examination of the arm, subcutaneous and other tumours were discovered, of an encephaloid and cancerous nature. The lungs, liver, and spleen were studded with purulent deposits. The venæ cavæ and lining membrane of the right side of the heart were inflamed.—*Ibid.*

